# Postnatal maintenance of the *5-Ht1a*-*Pet1* autoregulatory loop by serotonin in the raphe nuclei of the brainstem

**DOI:** 10.1186/1756-6606-7-48

**Published:** 2014-06-28

**Authors:** Ji-Young Kim, Ana Kim, Zhong-Qiu Zhao, Xian-Yu Liu, Zhou-Feng Chen

**Affiliations:** 1 Center for the Study of Itch, Washington University School of Medicine, St. Louis 63110, USA; 2 Departments of Anesthesiology, Washington University School of Medicine, St. Louis 63110, USA; 3 Developmental Biology, Washington University School of Medicine, St. Louis 63110, USA; 4 Psychiatry, Washington University School of Medicine, St. Louis 63110, USA

**Keywords:** Mouse, Serotonin, 5-HT1A, Pet1, Tph2, Development, Autoregulatory loop

## Abstract

**Background:**

Despite the importance of 5-HT1A as a major target for the action of several anxiolytics/antidepressant drugs, little is known about its regulation in central serotonin (5-hydroxytryptamine, 5-HT) neurons.

**Results:**

We report that expression of 5-HT1A and the transcription factor *Pet1* was impaired in the rostral raphe nuclei of mice lacking tryptophan hydroxylase 2 (*Tph2*) after birth. The downregulation of *Pet1* was recapitulated in *5-Ht1a*^*-/-*^ mice. Using an explant culture system, we show that reduction of *Pet1* and 5-HT1A was rescued in *Tph2*^*-/-*^ brainstem by exogenous 5-HT. In contrast, 5-HT failed to rescue reduced expression of *Pet1* in *5-Ht1a*^*-/-*^ brainstem explant culture.

**Conclusions:**

These results suggest a causal relationship between 5-HT1A and *Pet1*, and reveal a potential mechanism by which 5-HT1A*-Pet1* autoregulatory loop is maintained by 5-HT in a spatiotemporal-specific manner during postnatal development. Our results are relevant to understanding the pathophysiology of certain psychiatric and developmental disorders.

## Background

Central serotonin (5-hydroxytryptamine, 5-HT) neurons are composed of a cluster of nine groups (B1-9) located in the raphe nuclei and in the reticular formation of the brainstem: The caudal serotoninergic neurons (B1-3) project to the spinal cord and modulate sensory transmission, whereas the rostral serotonergic neurons (B4-9) project to the brain and modulate a wide spectrum of behaviors [1,2]. The development of the central 5-HT system in rodents is subjected to a highly coordinated regulation of both intrinsic and extrinsic factors [3,4]. After the establishment of the central serotonergic system, a matured neurotransmitter phenotype manifested by the persistent expression of a myriad of transcription factors, enzymes, and synaptic proteins is maintained through mechanisms that are yet to be elucidated. Conceivably, an impaired maintenance of 5-HT neuronal phenotype may contribute to abnormal physiologic function and the etiology of psychiatric disorders such as depression, anxiety and schizophrenia [5-9].

Molecular and genetic studies of mouse mutants have identified several transcription factors important for the specification and acquisition of the central 5-HT neuronal phenotype [10-12]. In postmitotic serotoninergic neurons, *Pet1*, *Gata3* and *Lmx1b* have been shown to regulate differentiation and survival of central 5-HT neurons because their deletion invariably results in a loss of 5-HT and the failure of 5-HT neurons to survive, either completely or partially [13-15]. On the other hand, the persistent expression of these transcription factors in serotonergic neurons during postnatal stages raises the possibility that they may be necessary for maintaining the 5-HT neurotransmitter phenotype during postnatal stage.

Serotonin exerts its function through a complex 5-HT receptor system comprised of at least 14 receptors, including several autoreceptors [16-18]. Many of 5-HT receptors show distinct spatiotemporal expression patterns in the brain, and the function of a 5-HT receptor may vary depending on where and when it is expressed. For example, 5-HT1A mediates anxiety behavior through its expression in the cortex during the early postnatal life rather than in adult stage [19,20]. A recent study has shown that altered expression of 5-HT1A autoreceptor without changing postsynaptic 5-HT1A has major impacts in physiological processes and animal behaviors [21]. Although overwhelming studies have been focused on 5-HT heteroreceptors, the role of 5-HT and its autoreceptors in the development and maintenance of central 5-HT neurons is poorly understood. We previously generated *Lmx1b*^*f/f/p*^ conditional knockout mice in which *Lmx1b* is specifically deleted in cells expressing transcription factor *Pet1,* a transcription factor exclusively expressed in central 5-HT neurons [22,23]. LMX1B is a LIM homoeodomain-containing transcription factor, which is required for the development of all central serotonergic neurons during embryogenesis [13,24]. Because loss of *Lmx1b* results in the loss of 5-HT as well as all central 5-HT neurons in *Lmx1b*^*f/f/p*^ mice at later stage [22], it is impossible to examine the requirement for 5-HT in the development of 5-HT neurons. To circumvent this problem, we have generated and analyzed mice lacking tryptophan hydroxylase 2 (TPH2), an enzyme essential for synthesizing 5-HT in the brain [25]. Although 5-HT appears to be dispensable for the development of central 5-HT neurons during embryogenesis, we found that it is important for maintaining the differentiated 5-HT neurotransmitter phenotype after birth.

## Results

### Normal generation and migration of presumptive central serotonergic neurons in *Tph2*^*-/-*^ mice

**Figure 1:**
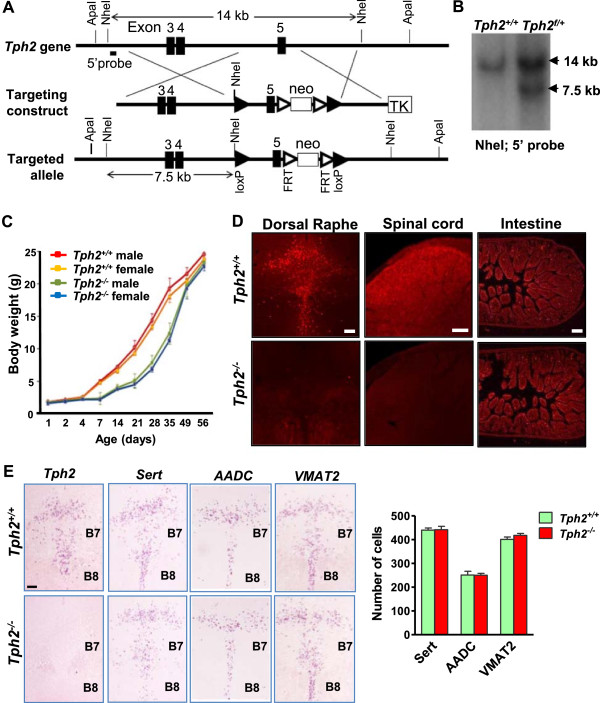
**Generation and characterization of *Tph2***^***-/- ***^**mice. (A)** Top: Schematic diagram of the genomic locus for the *Tph2* gene. Middle: the gene-targeting construct. Bottom: targeted *Tph2* allele. The black box denotes the coding exon. **(B)** Southern blot analysis of wild-type (*Tph2*^+/+^) and targeted ES clone (*Tph2*^*f/+*^). Predicted size of the genomic fragments was detected. **(C)** Growing curve of male and female *Tph2*^+/+^ and *Tph2*^*-/-*^ mice from P1 to 8-week. **(D)** Anti-5-HT immunoreactivity in the dorsal raphe (left), spinal cord (middle) and intestine (right) of *Tph2*^+/+^ mice (upper row) and *Tph2*^*-/-*^ mice (lower row). **(E)** Images (left) and quantified data (right) to show *in situ* hybridization of *Tph2*, *Sert, AADC* and *VMAT2* in *Tph2*^+/+^ and *Tph2*^*-/-*^ mice. Error bars represent SEM. n = 5. Scale bars, 100 μm.

To generate *Tph2*^*-/-*^ mice, two *loxP* sites were inserted to flank the exon 5 that encodes eukaryotic tryptophan hydroxylase domain essential for 5-HT synthesis (Figure 1A). The deletion of the *Tph2* exon 5 would generate a truncated non-functional protein. After electroporation with the targeting vector, several targeted ES cell clones were identified, and the germline transmission was obtained (Figure 1B). *Actb* cre mice were mated with *Tph2*^*f/+*^ mice to inactivate *Tph2* in the germline, and *Tph2*^*+/-*^ and *Tph2*^*-/-*^ mice were born at the expected mendelian ratio. *Tph2*^*-/-*^ mice showed decreased body weight and size relative to wild-type littermates beginning at postnatal day 6 (P6) and by 6 weeks of age all mutant mice showed normal body weight (Figure 1C). The body weight increase profile of our mutant mice is consistent with a recent study [26], but different from another report [27]. All *Tph2*^*-/-*^ mice in C57BL/6/129 background survived to the adulthood. Immunohistochemical analysis revealed no presence of 5-HT in the raphe nuclei and spinal cord of *Tph2*^*-/-*^ mice, whereas expression of 5-HT in the peripheral tissues was normal (Figure 1D). The absence of *Tph2* expression in *Tph2*^*-/-*^ mice was confirmed (Figure 1E). These data demonstrated that *Tph2* is the sole enzyme responsible for 5-HT synthesis in the brain, consistent with other reports [28-30]. To determine whether a loss of 5-HT may impair 5-HT neuronal differentiation, several molecular markers were examined in wild-type and *Tph2*^*-/-*^ mice. *In situ* hybridization studies of serotonin transporter (*Sert*) [31] revealed neither the expression pattern nor the number of *Sert*^*+*^ cells in the rostral raphe nuclei in *Tph2*^*-/-*^ mice was affected by the absence of 5-HT (Figure 1E). Expression of the aromatic L-amino acid decarboxylase gene (*AADC*) and vesicular monoamine transporter (*VMAT2*) was also comparable between wild-type and *Tph2*^*-/-*^ hindbrain (Figure 1E). These results indicated the normal presence of presumptive 5-HT neurons in *Tph2*^*-/-*^ mice, and 5-HT is required neither for the migration nor for the survival of central 5-HT neurons, in line with recent findings that showed normal expression of differentiation markers in *Tph2*^*-/-*^ mice [27].

### Downregulation of *Pet1* and *Gata3* expression in the rostral raphe nulcei of *Tph2*^*-/-*^ mice

**Figure 2:**
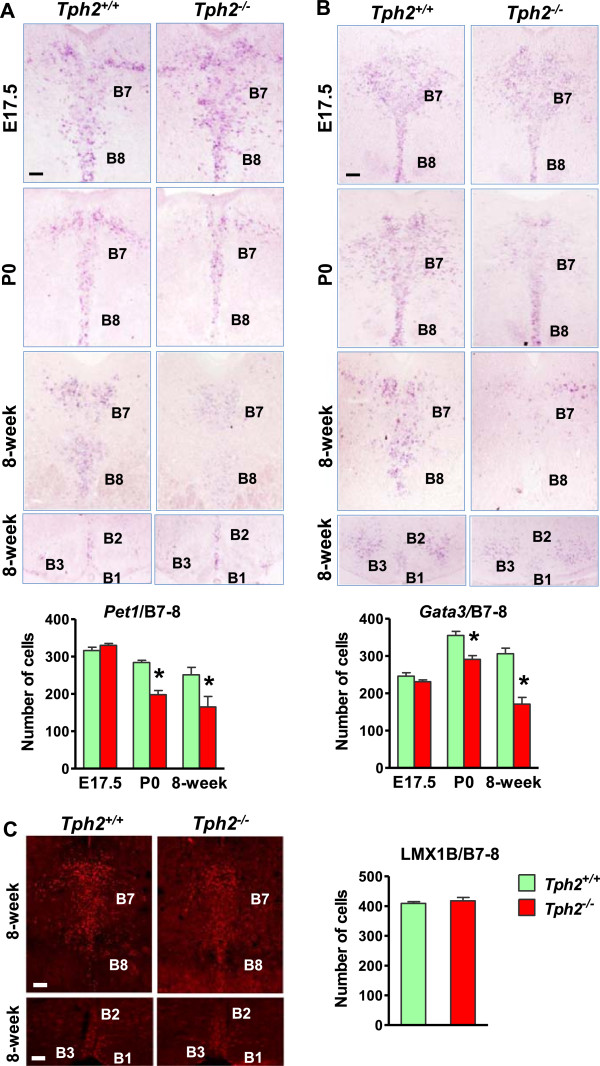
**The expression of *Pet1 *and *Gata3*, but not LMX1B was decreased in the dorsal raphe nuclei of *Tph2***^***-/- ***^**mice. (A and B)** Expression of *Pet1***(A)** and *Gata3***(B)** detected by *in situ* hybridization in the dorsal and caudal raphe nuclei of *Tph2*^+/+^ mice and *Tph2*^*-/-*^ mice at embryonic day 17.5 (E17.5), postnatal day 0 (P0) and adult stages. **(C)** Immunohistochemistry images and quantified data to show LMX1B expression in the dorsal and caudal raphe nuclei of adult *Tph2*^+/+^ mice and *Tph2*^*-/-*^ mice. Error bars represent SEM. n = 6. *P < 0.05, versus *Tph2*^+/+^, unpaired *t* test. Scale bars, 100 μm.

To further analyze the differentiation phenotype of presumptive 5-HT neurons, we examined the expression of transcription factors in the raphe nuclei of adult *Tph2*^*-/-*^ mice during embryonic and early postnatal development. At E17.5, *Pet1* and *Gata3* expression was indistinguishable between wild-type and *Tph2*^*-/-*^ hindbrain (Figures 2A, B, E17.5). Beginning at P0 the expression of *Pet1* and *Gata3* was significantly reduced in the rostral raphe nuclei of *Tph2*^*-/-*^ mice compared with wild-type mice (Figures 2A, B, P0), which persisted at adult stage (Figure 2A, B, 8-week). The expression of *Pet1* and *Gata3* remains unchanged in the caudal raphe nuclei (B1-3) of *Tph2*^*-/-*^ mice (Figure 2A, B). The expression pattern of LMX1B and the number of LMX1B^+^ cells were similar between wild-type and *Tph2*^*-/-*^ mice (Figure 2C).

### Reduced expression of *Pet1* but not *Gata3* in *5-Ht1a*^*-/-*^ mice

**Figure 3:**
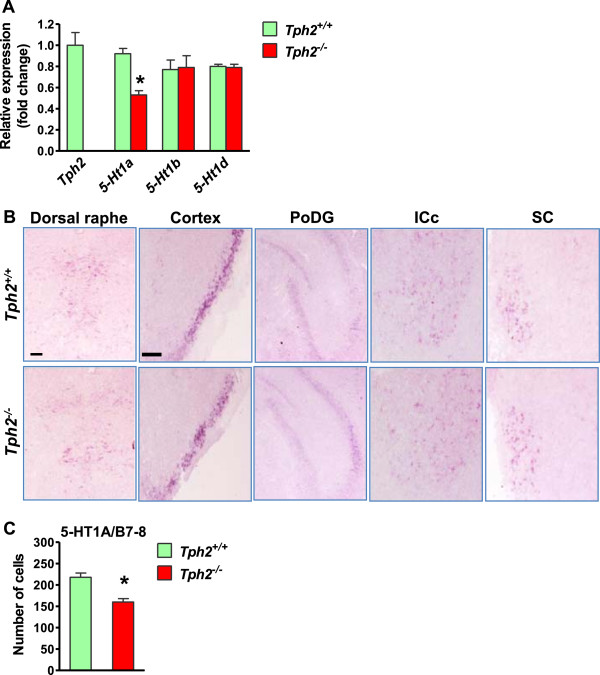
**5-HT1A expression was decreased in the dorsal raphe nuclei of *Tph2***^***-/- ***^**mice. (A)** qRT-PCR analysis of *5Ht1a, 5Ht1b* and *5Ht1d* in *Tph2*^+/+^ and *Tph2*^*-/-*^ hindbrain. *Tph2* mRNA level in the *Tph2*^*-/-*^ hindbrain was used as a negative control. Relative expression was quantified using 18S rRNA as reference gene. **(B)** Immunostaining of 5-HT1A in the dorsal raphe nucleus, cortex, polymorph layer dentate gyrus (PoDG), inferior colliculus central nucleus (ICc) and superior colliculus (SC) of *Tph2*^+/+^ mice and *Tph2*^*-/-*^ mice. **(C)** Quantification of 5-HT1A^+^ neurons in B7-8 raphe nucleus. Error bars represent SEM. n = 6. *P < 0.05, versus *Tph2*^+/+^, unpaired *t* test. Scale bars, 100 μm.

The downregulation of *Pet1* and *Gata3* in *Tph2*^*-/-*^ mice suggests that their normal expression may be maintained by 5-HT via 5-HT autoreceptors. To examine this possibility, we first compared expression level of three 5-HT autoreceptors, *5-Ht1a, 5-Ht1b* and *5-Ht1d,* in the hindbrain of *Tph2*^*+/+*^ mice and *Tph2*^*-/-*^ mice using real-time RT-PCR (qRT-PCR). *5-Ht1a* expression was significantly reduced in *Tph2*^*-/-*^ hindbrain relative to the control, while the expression of *5-Ht1b* and *5-Ht1d* was unaltered (Figure 3A). Immunostaining of 5-HT1A showed a reduction in the number of 5-HT1A^+^ cells (~25.7%) in B7-8 nuclei of *Tph2*^*-/-*^ mice compared with wild-type mice (Figure 3B, C). The expression of 5-HT1A was not affected in other brain regions of the *Tph2*^*-/-*^ mice (Figure 3B). These results suggest that 5-HT is involved in the regulation of 5-HT1A expression in a highly specific manner.

**Figure 4:**
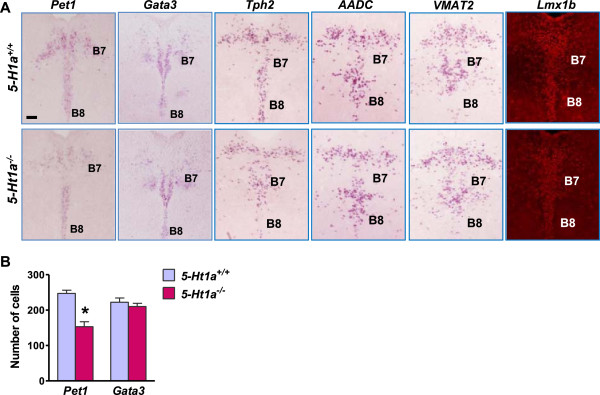
**The expression of *Pet1 *but not *Gata3 *was decreased in *5Ht1a***^***-/- ***^**mice. (A)***In situ* hybridization images to show the expression of *Pet1, Gata3, Tph2, AADC* and *VMAT2* and immunostaining of LMX1B in the dorsal raphe nuclei of *5-Ht1a*^*+/+*^ mice (upper row) and *5-Ht1a*^*-/-*^ mice (lower row). **(B)** Quantification of positive cells for *Pet1* and *Gata3* in B7-8 raphe nuclei. Error bars represent SEM. n = 6. *P < 0.05, versus *5-Ht1a*^*+/+*^, unpaired *t* test. Scale bar, 100 μm.

We next asked whether the maintenance of *Pet1* and *Gata3* expression depends on 5-HT1A by examining their expression in *5-Ht1a*^*-/-*^ mice [32]. *Pet1* was significantly diminished in *5-Ht1a*^*-/-*^ mice relative to wild-type mice at P0 (approximately ~29.8% loss of *Pet1* in B7-B8 nuclei) (Figure 4). Interestingly, *Gata3* expression was normal in the raphe nuclei of *5-Ht1a*^*-/-*^ mice (Figure 4), and so were *Tph2*, *AADC, VMAT2* and LMX1B (Figure 4A). These findings suggest that 5-HT can regulate *Pet1*, but not *Gata3*, through *5-Ht1a* in the rostral raphe nuclei.

### Exogenous 5-HT rescued the expression of *Pet1* and *5-Ht1a* in *Tph2*^*-/-*^ brainstem

**Figure 5:**
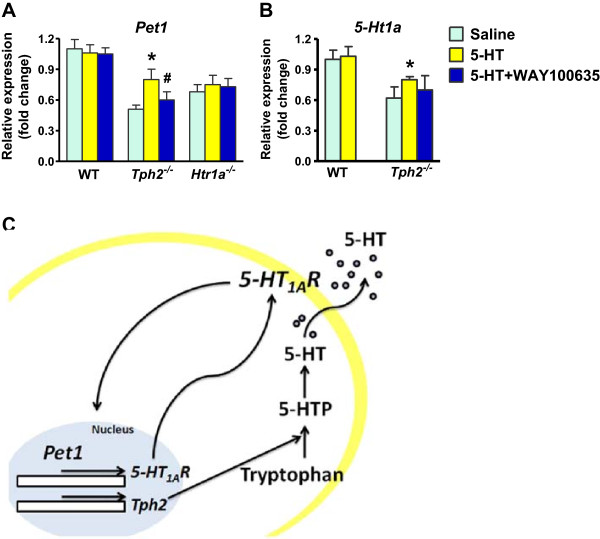
**The regulation of *Pet1 *and *5-Ht1a *expression by 5-HT in brainstem explants. (A and B)** The hindbrain explants of *Tph2*^*-/-*^ mice, *5-Ht1a*^*-/-*^ mice and their littermates wild-type mice were treated with 5-HT either alone or in combination with WAY-100635, and the expression level of *Pet1***(A)** and *5-Ht1a***(B)** was measured using qRT-PCR. Relative expression was quantified using 18S rRNA as reference gene. Error bars represent SEM. n = 5. *P < 0.05, versus Saline and ^#^*P* < 0.05, versus 5-HT, one-way ANOVA followed by *post hoc* analysis. **(C)** Diagram to show a 5-HT-dependent *5-Ht1a/Pet1* auto regulatory feedback loop.

To further test this possibility, we asked whether the downregulation of *Pet1* in *Tph2*^*-/-*^ hindbrain would be rescued by 5-HT in *Tph2*^*-/-*^ mice, and if so, whether this rescue could be blocked by the 5-HT1A antagonist. Indeed, qRT-PCR analysis of *Pet1* expression showed that the reduced expression of *Pet1* was partially rescued in *Tph2*^*-/-*^ hindbrain explants by 5-HT (Figure 5A). Importantly, this rescued effect was abolished with the treatment of WAY-100635, a potent selective 5-HT1A antagonist [33]. In contrast, in *5-Ht1a*^*-/-*^ hindbrain explants, the expression level of *Pet1* was neither significantly altered by 5-HT nor by a combined treatment of 5-HT and WAY-100635 (Figure 5A). Together, these results provide further evidence suggesting that 5-HT regulates *Pet1* expression via *5-Ht1a*.

We next assessed whether downregulation of *5-Ht1a* expression in *Tph2*^*-/-*^ mice could be rescued by 5-HT using the explant culture. qRT-PCR analysis showed a significant increase in *5-Ht1a* expression in *Tph2*^*-/-*^ hindbrain explants after treatment with 5-HT compared with the saline treatment (Figure 5B). Our data thus support the role of 5-HT in regulating *5-Ht1a* expression.

## Discussion

5-HT has long been postulated as an important signaling molecule necessary for regulating multiple developmental processes such as neurogenesis, differentiation, and survival of central 5-HT neurons [34,35]. Although several recent studies have reported the physiological and developmental consequences resulting from an ablation of central serotonin by genetic deletion of *Tph2* gene [27-30], definite *in vivo* evidence for a developmental role of 5-HT is still lacking. In this study, we found that mice lacking central 5-HT exhibited normal position and numbers of presumptive 5-HT neurons in the raphe nuclei, indicating that 5-HT is dispensable for their migration and survival. Nonetheless, the early postnatal expression of *Pet1*, *Gata3* and *5-Ht1a* was significantly impaired in the mutants. Our studies thus for the first time uncover an important function of 5-HT in the maintenance of differentiation program of central 5-HT neurons.

Which receptor mediates the function of 5-HT to maintain the differentiated 5-HT neuronal phenotype? Several observations indicate that 5-HT functions via 5-HT1A to regulates *Pet1,* which in turn may control an appropriate expression of 5-HT1A in 5-HT neurons. First, *Pet1* is significantly reduced in the rostral raphe nuclei of *Tph2*^*-/-*^ mice, and *5-Ht1a*^*-/-*^ mice recapitulate this phenotype. Second, decreased expression of *Pet1* in *Tph2*^*-/-*^ mice was rescued by 5-HT treatment, whereas the same treatment failed to rescue *Pet1* reduction in *5-Ht1a*^*-/-*^ mice. Third, previous studies identified at least one *Pet1* binding site in the regulatory regions of both human and mouse *5-Ht1a*[23,36], suggesting that *Pet1* can also act upstream of *5-Ht1a*. It will be interesting to examine whether *5-Ht1a* expression is regulated by *Pet1* using *Pet1* mutant mice. However, addressing this issue would be complicated by the possibility that *Pet1* may regulate *Tph2* (thus 5-HT level) and *5-Ht1a* simultaneously. Our results are different from several recent studies which showed that expression of *5-Ht1a* autoreceptor in dorsal raphe nuclei was slightly increased in *Tph2*^*-/-*^ mice [27] or remained the same [37]. Several factors may contribute to the discrepancies. First, different methods that have been employed (quantitative autoradiography vs. ISH and qRT-PCR). Second, different lines of *Tph2* knockout mice may vary due to different genetic backgrounds. The postnatal growth profile of our *Tph2*^*-/-*^ mice is reminiscent of the one generated by another lab [30], with a catch-up in body weight at adult stage. In contrast, another line of *Tph2*^*-/-*^ mice exhibited lean body weight, even at the adult stage [27]. Finally, conditional *Tph2*^*-/-*^ mice showed low level of 5-HT due to incomplete deletion of *Tph2*[37]. Our *Tph2*^*-/-*^ mice completely lack 5-HT in the brainstem. In light of these differences, behavioral phenotype of various lines of *Tph2* mutant mice ought to be carefully interpreted, given that expression of *5-ht1a* autoreceptor is very sensitive to the 5-HT level. The normal growth of *Tph2*^*-/-*^ mice affords distinct advantage because exogenous administration of 5-HTP could fully restore a behavioral phenotype [38], thereby attributing the phenotype to lack of 5-HT instead of developmental changes. Recent analysis of these mutant mice also shed light on the role of 5-HT in the reward system [39]. Nevertheless, the present data provide the genetic, molecular and pharmacological evidence suggesting that *5-Ht1a* and *Pet1* may act in an autoregulatory feedback pathway, whose integrity is dependent on 5-HT (Figure 5C).

One notable finding is that the 5-HT-dependent maintenance program exhibits a highly regulated spatiotemporal specificity: its requirement is restricted only to postnatal rostral raphe nuclei. Given that distinct genetic mechanisms are likely to be recruited to coordinate spatiotemporal development of different subtypes of 5-HT neurons [40], the observation that the requirement for 5-HT-mediated transcriptional regulation is restricted to rostral raphe nuclei may underlie the heterogeneity of central 5-HT neurons. Our results suggest that the maintenance of the maturation and differentiation of 5-HT neurons invoke 5-HT-dependent and -independent molecular machineries. This heterogeneity is also manifested at transcriptional level. For example, we show that postnatal maintenance of *Lmx1b* is independent of *Pet1* and *Gata3*, whereas *Gata3* expression is independent of *Pet1* in the rostral raphe nuclei. It is surprising that an impaired expression of *Pet1* in postnatal *5-Ht1a*^*-/-*^ mice did not affect expression of several *Pet1* binding site-containing terminal differentiation genes (e.g. *Sert* and *VMAT2*) which were downregulated in *Pet1*^*-/-*^ mice [14]. Perhaps other transcription factors such as *Lmx1b* may be required. Alternatively, a complete rather than a partial elimination of *Pet1* may be necessary to reveal a full transcriptional requirement in the regulation of downstream effectors. The finding that *Gata3* is down-regulated by the absence of 5-HT but not by 5-HT1A also suggests that several 5-HT autoreceptors coordinate to regulate transcription factor expression.

5-HT1A has emerged as a major target for pharmacological intervention against a wide spectrum of psychiatric diseases [41-43]. In addition to its postsynaptic action, activation of 5-HT1A on the soma and dendrites of 5-HT neurons has been shown to inhibit the release of 5-HT as well as the firing rate of 5-HT neurons [44,45]; Conversely, blockage or desensitization of 5-HT1A in 5-HT neurons enhances the firing rate of 5-HT neurons and/or the 5-HT signaling, a mechanism suggested to be partly underlie the actions of anxiolytic and antidepressant drugs [46-48]. Importantly, a recent study showed that about 30% decrease of 5-HT1A in the raphe nuclei increased the firing rate of 5-HT neurons and subsequently impacted several aspects of animal behaviors including the responses of animals to antidepressants [21]. Our studies suggest that a normal 5-HT homeostasis is necessary for a full expression of 5-HT1A in the rostral raphe nuclei of the brainstem. An altered *5-Ht1a*/*Pet1* expression and regulation may impact other 5-HT autoreceptors and thereby contribute to the development of mood disorders (e.g. major depressive disorder, aggression and anxiety) [21,42,46,47,49,50]. In this context, the identification of a 5-HT-dependent *5-Ht1a/Pet1* autoregulatory feedback loop (Figure 5C) required for maintaining appropriate 5-HT signaling and homeostasis in postnatal raphe nuclei is of important clinical implications for designing target-specific therapeutic intervention of these disorders.

## Methods

### Animals

*5-Ht1a* KO mice and *Actb-cre* mice were genotyped as previously described [32,51]. Experimental procedures were conducted in accordance with policies of the National Institutes of Health and were approved by the Animal Studies Committee at Washington University School of Medicine.

### Generation and genotyping of *Tph2*^*-/-*^ mice

*Tph2* targeting vector was generated by bacterial recombineering approach as previously described [52]. Mouse genomic 129/SvJ DNA was obtained from Sanger Institute (UK). The PGK-neomycin-resistance (neo) and thymidine kinase (TK) cassette was inserted as a positive and negative drug selection marker. The linearized targeting plasmid was electroporated into AB1 ES cells. Correctly targeted ES cell clones identified by Southern blot analysis using external probes were injected into C57BL/6 blastocysts to generate chimeric mice. Male chimeras were mated with wild-type females to produce heterozygous mice. The primers used for PCR genotyping were 5′-GCAGCCAGTAGACGTCTCTTAC-3′, 5′-ACCACATTGACATACGGGTC-3′ and 5′-TGGGGCCTGCCGATAGTAACAC-3′ and 420 bp wild-type and 640 bp mutant bands were produced, respectively.

### Immunohistochemistry and *in situ* hybridization

Immunohistochemical staining and *in situ* hybridization were performed as previously described [22]. Briefly, mice were anesthetized with an overdose of ketamine, and fixed by intracardiac perfusion with cold PBS (0.01 M, pH 7.4) followed by 4% paraformaldehyde. The brain, spinal cord and small intestine were immediately removed, postfixed in the same fixative overnight at 4°C and cryoprotected in 30% sucrose solution. Frozen tissue was sectioned at 20 μm thickness using a cryostat. Free-floating sections were blocked in a solution containing 2% donkey serum and 0.3% Triton X-100 in PBS for 1 h at room temperature. The sections were incubated with rabbit anti-5-HT (1:5,000, Immunostar), rabbit anti-5-HT1A (1:200, Santa Cruz) or rabbit anti-LMX1B (1:1,000,) antibodies overnight at 4°C followed by Cy3-conjugated secondary antibodies (Jackson ImmunoResearch). For the *in situ* hybridization study, a digoxigenin-labeled cRNA probe was used as described earlier [22]. Images were taken using a Nikon Eclipse Ti-U microscope. Images of wild-type and knock-out mice were overlaid with the same plate and box of identical size was centered on a template image of the comparable rostrocaudal levels using the ventricle and midline nuclei as the reference point. All clearly visible cells with positive signals within this box were quantified using image J software. Four tissue sections per B nucleus were analyzed in each mouse.

### qRT-PCR analysis

qRT-PCR was performed as previously described [53]. The defined area of the hindbrain was dissected out, and total RNA was isolated and genomic DNA was removed in accordance with manufacturer’s instructions (RNeasy plus mini kit; QIAGEN). Single strand cDNA was synthesized from RNA samples (2 μg) using SuperScriptII RT-PCR kit (Life Technologies) in reverse transcription reaction. Gene expression was determined by real-time PCR (StepOnePlus; Applied Biosystems). The primers used are:

*Tph2* (NM_173391.3): 5′- GAGCTTGATGCCGACCAT-3′; 5′- TGGCCACATCCACAAAATAC-3′; amplicon size: 76 nt.

*5-Ht1a* (NM_008308.4): 5′-GCCCTGGAAGCAACACCTAA-3′; 5′-CTGCAAAAAGCACTGTCCCC -3′; amplicon size: 115 nt.

*5-Ht1b* (NM_010482.1): 5′-TCGTTGCCACCCTTCTTCTG-3′; 5′-CGTGGTCGGTGTTCACAAAG-3′; amplicon size: 76 nt.

*5-Ht1d* (NM_008309.4): 5′-GGGTCAATTCATCAAGAACACA-3′; 5′-GCTTGGAAGCTCTGAGGTGT-3′; amplicon size: 93 nt.

18S rRNA (NR_003278.3): 5′-AAACGGCTACCACATCCAAG -3′; 5′-CCTCCAATGGATCCTCGTTA -3′; amplicon size: 155 nt.

Real-time PCR was carried out with FastStart Universal SYBR Green Master (Roche Applied Science). All samples (0.1 μl) were assayed in duplicates. Thermal cycling was initiated with denaturation at 95°C for 10 min. After this initial step, 40 cycles of PCR (heating at 95°C for 10 s and 60°C for 30 s) were performed. Data were analyzed using Comparative CT Method (StepOne Software v2.2.2.) and the expression of target mRNA was normalized to the expression of 18S rRNA.

### Hindbrain explant culture

Hindbrain explant culture was performed as described [54]. Briefly, hindbrains from wild-type and knock-out mice at P0 were dissected out in cold Leibovitz’s medium (L15; Invitrogen) with 5% calf serum (Life Technologies). The explants were cultured for 1 h at 37°C in Neurobasal medium (Life Technologies) containing 1% calf-serum (Life Technologies), 0.5% B27 supplement (Life Technologies), 2 mM glutamine (Sigma) and 1% penicillin/streptomycin (Life Technologies). Subsequently, the medium was replaced with serum-free medium and the explants were treated with 10 μM of 5-HT or 5-HT1A antagonist WAY-100635 for 6 h. For the use of combination of WAY-100635 and 5-HT, the explants were pre-treated with WAY-100635 for 2 h before addition of 5-HT. 5-HT and WAY-100635 were dissolved in saline and the group with the saline treatment was used as the vehicle control.

### Statistical analysis

All data are presented as mean ± SEM and analyses were performed using Prism (version 5.03 GraphPad Software, Inc). Experimental groups were compared by unpaired two-tailed Student’s *t*-test or one-way ANOVA of variance with *post hoc* analysis. *P* < 0.05 was considered as statistically significant.

## Abbreviations

5-HT: 5-Hydroxytryptamine; TPH2: Tryptophan hydroxylase 2; SERT: Serotonin transporter; AADC: Aromatic L-amino acid decarboxylase gene; VMAT2: Vesicular monoamine transporter; qRT-PCR: Real-time reverse transcription polymerase chain reaction; TK: Thymidine kinase.

## Competing interests

The authors declare that they have no competing interests.

## Authors’ contributions

JYK generated floxed *Tph2* mutant mice and performed experiments, AK, ZQZ, and XYL participated in experiments, organization of data and preparation of the manuscript. JYK and ZFC wrote the paper. All authors read and approved the final manuscript.
